# Understanding the Influence of Race/Ethnicity, Gender, and Class on Inequalities in Academic and Non-Academic Outcomes among Eighth-Grade Students: Findings from an Intersectionality Approach

**DOI:** 10.1371/journal.pone.0141363

**Published:** 2015-10-27

**Authors:** Laia Bécares, Naomi Priest

**Affiliations:** 1 Centre on Dynamics of Ethnicity, Department of Social Statistics, University of Manchester, Manchester, United Kingdom; 2 Australian National University, Acton, Australia; Kyoto University, JAPAN

## Abstract

Socioeconomic, racial/ethnic, and gender inequalities in academic achievement have been widely reported in the US, but how these three axes of inequality intersect to determine academic and non-academic outcomes among school-aged children is not well understood. Using data from the US Early Childhood Longitudinal Study—Kindergarten (ECLS-K; N = 10,115), we apply an intersectionality approach to examine inequalities across eighth-grade outcomes at the intersection of six racial/ethnic and gender groups (Latino girls and boys, Black girls and boys, and White girls and boys) and four classes of socioeconomic advantage/disadvantage. Results of mixture models show large inequalities in socioemotional outcomes (internalizing behavior, locus of control, and self-concept) across classes of advantage/disadvantage. Within classes of advantage/disadvantage, racial/ethnic and gender inequalities are predominantly found in the most advantaged class, where Black boys and girls, and Latina girls, underperform White boys in academic assessments, but not in socioemotional outcomes. In these latter outcomes, Black boys and girls perform better than White boys. Latino boys show small differences as compared to White boys, mainly in science assessments. The contrasting outcomes between racial/ethnic and gender minorities in self-assessment and socioemotional outcomes, as compared to standardized assessments, highlight the detrimental effect that intersecting racial/ethnic and gender discrimination have in patterning academic outcomes that predict success in adult life. Interventions to eliminate achievement gaps cannot fully succeed as long as social stratification caused by gender and racial discrimination is not addressed.

## Introduction

The US racial/ethnic academic achievement gap is a well-documented social inequality [1]. National assessments for science, mathematics, and reading show that White students score higher on average than all other racial/ethnic groups, particularly when compared to Black and Hispanic students [2, 3]. Explanations for these gaps tend to focus on the influence of socioeconomic resources, neighborhood and school characteristics, and family composition in patterning socioeconomic inequalities, and on the racialized nature of socioeconomic inequalities as key drivers of racial/ethnic academic achievement gaps [4–10]. Substantial evidence documents that indicators of socioeconomic status, such as free or reduced-price school lunch, are highly predictive of academic outcomes [2, 3]. However, the relative contribution of family, neighborhood and school level socioeconomic inequalities to racial/ethnic academic inequalities continues to be debated, with evidence suggesting none of these factors fully explain racial/ethnic academic achievement gaps, particularly as students move through elementary school [11]. Attitudinal outcomes have been proposed by some as one explanatory factor for racial/ethnic inequalities in academic achievement [12], but differences in educational attitudes and aspirations across groups do not fully reflect inequalities in academic assessment. For example, while students of poorer socioeconomic status have lower educational aspirations than more advantaged students [13], racial/ethnic minority students report higher educational aspirations than White students, particularly after accounting for socioeconomic characteristics [14–16]. Similarly, while socio-emotional development is considered highly predictive of academic achievement in school students, some racial/ethnic minority children report better socio-emotional outcomes than their White peers on some indicators, although findings are inconsistent [17–22].

In addition to inequalities in academic achievement, racial/ethnic and socioeconomic inequalities also exist across measures of socio-emotional development [23–26]. And as with academic achievement, although socioeconomic factors are highly predictive of socio-emotional outcomes, they do not completely explain racial/ethnic inequalities in school-related outcomes not focused on standardized assessments [11].

Further complexity in understanding how academic and non-academic outcomes are patterned by socioeconomic factors, and how this contributes to racial/ethnic inequalities, is added by the multi-dimensional nature of socioeconomic status. Socioeconomic status is widely recognized as comprising diverse factors that operate across different levels (e.g. individual, household, neighborhood), and influence outcomes through different causal pathways [27]. The lack of interchangeability between measures of socioeconomic status within and between levels (e.g. income, education, occupation, wealth, neighborhood socioeconomic characteristics, or past socioeconomic circumstances) is also well established, as is the non-equivalence of measures between racial/ethnic groups [27]. For example, large inequalities have been reported across racial/ethnic groups within the same educational level, and inequalities in wealth have been shown across racial/ethnic that have similar income. It is therefore imperative that studies consider these multiple dimensions of socioeconomic status so that critical social gradients across the entire socioeconomic spectrum are not missed [27], and racial/ethnic inequalities within levels of socioeconomic status are adequately documented. It is also important that differences in school outcomes are considered across levels of socioeconomic status within and between racial/ethnic groups, so that the influence of specific socioeconomic factors on outcomes within specific racial/ethnic groups can be studied [28]. However, while these analytic approaches have been identified as research priorities in order to enhance our understanding of the complex ways in which socioeconomic status and race/ethnicity intersect to influence school outcomes, research that operationalizes these recommendations across academic and non-academic outcomes of school children is scant.

In addition to the complexity that arises from race/ethnicity, socioeconomic status, and intersections between them, different patterns in academic and non-academic outcomes by gender have also received longstanding attention. Comparisons across gender show that, on average, boys have higher scores in mathematics and science, whereas girls have higher scores in reading [2, 3, 29]. In contrast to explanations for socioeconomic inequalities, gender differences have been mainly attributed to social conditioning and stereotyping within families, schools, communities, and the wider society [30–35]. These socialization and stereotyping processes are also highly relevant determining factors in explaining racial/ethnic academic and non-academic inequalities [35, 36], as are processes of racial discrimination and stigmatization [37, 38]. Gender differences in academic outcomes have been documented as differently patterned across racial/ethnic groups and across levels of socioeconomic status. For example, gender inequalities in math and science are largest among White and Latino students, and smallest among Asian American and African American students [39–43], while gender gaps in test scores are more pronounced among socioeconomically disadvantaged children [44, 45]. In terms of attitudes towards math and sciences, gender differences in attitudes towards math are largest among Latino students, but gender differences in attitudes towards science are largest among White students [39, 40]. Gender differences in socio-developmental outcomes and in non-cognitive academic outcomes, across race/ethnicity and socio-economic status, have received far less attention; studies that consider multiple academic and non-academic outcomes among school aged children across race/ethnicity, socioeconomic status and gender are limited in the US and internationally.

Understanding how different academic and non-academic outcomes are differently patterned by race/ethnicity, socio-economic status, and gender, including within and between group differences, is an important research area that may assist in understanding the potential causal pathways and explanations for observed inequalities, and in identifying key population groups and points at which interventions should be targeted to address inequalities in particular outcomes [28, 46]. Not only is such knowledge critical for population level policy and/or local level action within affected communities, but failing to detect potential factors for interventions and potential solutions is argued as reinforcing perceptions of the unmodifiable nature of inequality and injustice [46].

Notwithstanding the importance of documenting patterns of inequality in relation to a particular social identity (e.g. race/ethnicity, gender, class), there is increasing acknowledgement within both theoretical and empirical research of the need to move beyond analyzing single categories to consider simultaneous interactions between different aspects of social identity, and the impact of systems and processes of oppression and domination (e.g., racism, classism, sexism) that operate at the micro and macro level [47, 48]. Such intersectional approaches challenge practices that isolate and prioritize a single social position, and emphasize the potential of varied inter-relationships of social identities and interacting social processes in the production of inequities [49–51]. To date, exploration of how social identities interact in an intersectional way to influence outcomes has largely been theoretical and qualitative in nature. Explanations offered for interactions between privileged and marginalized identities, and associated outcomes, include family and teacher socialization of gender performance (e.g. math and science as male domains, verbal and emotional skills as female), as well as racialized stereotypes and expectations from teachers and wider society regarding racial/ethnic minorities that are also gendered (e.g. Black males as violent prone and aggressive, Asian females as submissive) [52–57]. That is, social processes that socialize and pattern opportunities and outcomes are both racialized and gendered, with racism and sexism operating in intersecting ways to influence the development and achievements of children and youth [58–60]. Socioeconomic status adds a third important dimension to these processes, with individuals of the same race/ethnicity and gender having access to vastly different resources and opportunities across levels of socioeconomic status. Moreover, access to resources as well as socialization experiences and expectations differ considerably by race and gender within the same level of socio-economic status. Thus, neither gender nor race nor socio-economic status alone can fully explain the interacting social processes influencing outcomes for youth [27, 28]. Disentangling such interactions is therefore an important research priority in order to inform intervention to address inequalities at a population level and within local communities.

In the realm of quantitative approaches to the study of inequality, studies often examine separate social identities independently to assess which of these axes of stratification is most prominent, and for the most part do not consider claims that the varied dimensions of social stratification are often juxtaposed [56, 61]. A pressing need remains for quantitative research to consider how multiple forms of social stratification are interrelated, and how they combine interactively, not just additively, to influence outcomes [46]. Doing so enables analyses that consider in greater detail the representation of the embodied positions of individuals, particularly issues of multiple marginalization as well as the co-occurrence of some form of privilege with marginalization [46]. It is important to note that the languages of statistical interaction and of intersectionality need to be carefully distinguished (e.g. intersectional additivity or additive assumptions, versus additive scale and cross-product interaction terms) to avoid misinterpretation of findings, and to ensure appropriate application of statistical interaction to enable the description of outcome measures for groups of individuals at each cross-stratified intersection [46]. Ultimately this will provide more nuanced and realistic understandings of the determinants of inequality in order to inform intervention strategies.

This study fills these gaps in the literature by examining inequalities across several eighth grade academic and non-academic outcomes at the intersection of race/ethnicity, gender, and socioeconomic status. It aims to do this by: identifying classes of socioeconomic advantage/disadvantage from kindergarten to eighth grade; then ascertaining whether membership into classes of socioeconomic advantage/disadvantage differ for racial/ethnic and gender groups; and finally, by contrasting academic and non-academic outcomes at the intersection of race/ethnicity, gender and socioeconomic advantage/disadvantage. Intersecting identities of race/ethnicity, gender, and socioeconomic characteristics are compared to the reference group of White boys in the most advantaged socioeconomic category, as these are the three identities (male, White, socioeconomically privileged) that experience the least marginalization when compared to racial/ethnic and gender minority groups in disadvantaged socioeconomic positions.

## Methods

### Data

This study used data on singleton children from the Early Childhood Longitudinal Study—Kindergarten (ECLS-K). The ECLS-K employed a multistage probability sample design to select a nationally representative sample of children attending kindergarten in 1998–99. In the base year the primary sampling units (PSUs) were geographic areas consisting of counties or groups of counties. The second-stage units were schools within sampled PSUs. The third- and final-stage units were children within schools [62]. Analyses were conducted on data collected from direct child assessments, as well as information provided by parents and school administrators.

### Ethics Statement

This article is based on the secondary analysis of anonymized and de-identified Public-Use Data Files available to researchers via the Inter-University Consortium for Political and Social Research (ICPSR). Human participants were not directly involved in the research reported in this article; therefore, no institutional review board approval was sought.

### Measures

#### Outcome Variables

Eight outcome variables, all assessed in eighth grade, were selected to examine the study aims: two measures relating to non-cognitive academic skills (perceived interest/competence in reading, and in math); three measures capturing socioemotional development (internalizing behavior, locus of control, self-concept); and three measures of cognitive skills (math, reading and science assessment scores).

For the eighth-grade data collection, children completed the 16-item Self Description Questionnaire (SDQ) II [63], where they provided self-assessments of their academic skills by rating their perceived competence and interest in English and mathematics. The SDQ also asked children to report on problem behaviors with which they might struggle. Three subscales were produced from the SDQ items: The SDQ Perceived Interest/Competence in Reading, including four items on grades in English and the child’s interest in and enjoyment of reading. The SDQ Perceived Interest/Competence in Math, including four items on mathematics grades and the child’s interest in and enjoyment of mathematics. And the SDQ Internalizing Behavior subscale, which includes eight items on internalizing problem behaviors such as feeling sad, lonely, ashamed of mistakes, frustrated, and worrying about school and friendships [62].

The Self-Concept and Locus of Control scales ask children about their self-perceptions and the amount of control they have over their own lives. These scales, adopted from the National Education Longitudinal Study of 1988, asked children to indicate the degree to which they agreed with 13 statements (seven items in the Self-Concept scale, and six items in the Locus of Control Scale) about themselves, including “I feel good about myself,” “I don’t have enough control over the direction my life is taking,” and “At times I think I am no good at all.” Responses ranged from “strongly agree” to “strongly disagree.” Some items were reversed coded so that higher scores indicate more positive self-concept and a greater perception of control over one’s own life. The seven items in the Self-Concept scale, and the six items in the Locus of Control were standardized separately to a mean of zero and a standard deviation of 1. The scores of each scale are an average of the standardized scores [62].

Academic achievement in reading, mathematics and science was measured with the eighth-grade direct cognitive assessment battery [62].

Children were given separate routing assessment forms to determine the level (high/low) of their reading, mathematics, and science assessments. The two-stage cognitive assessment approach was used to maximize the accuracy of measurement and reduce administration time by using the child’s responses from a brief first-stage routing form to select the appropriate second-stage level form. First, children read items in a booklet and recorded their responses on an answer form. These answer forms were then scored by the test administrator. Based on the score of the respective routing forms, the test administrator then assigned a high or low second-stage level form of the reading and mathematics assessments. For the second-stage level tests, children read items in the assessment booklet and recorded their responses in the same assessment booklet. The routing tests and the second-stage tests were timed for 80 minutes [62]. The present analyses use the standardized scores (T-scores), allowing relative comparisons of children against their peers.

#### Individual and Contextual Disadvantage Variables

Latent Class Analysis, described in greater detail below, was used to classify students into classes of individual and contextual advantage or disadvantage. Nine constructs, measuring characteristics at the individual-, school-, and neighborhood-level, were captured using 42 dichotomous variables measured across the different waves of the ECLS-K.

Individual-level variables captured household composition, material disadvantage, and parental expectations of the children’s success. Measures included whether the child lived in a single-parent household at kindergarten, first, third, fifth and eighth grades; whether the household was below the poverty threshold level at kindergarten, fifth and eighth grades; food insecurity at kindergarten, first, second and third grades; and parental expectations of the child’s academic achievement (categorized as up to high school and more than high school) at kindergarten, first, third, fifth and eighth grades. An indicator of whether parents had moved since the previous interview (measured at kindergarten, first, third, fifth and eighth grades) was included to capture stability in the children’s life. A household-level composite index of socioeconomic status, derived by the National Center for Education Statistics, was also included at kindergarten, first, third, fifth and eighth grades. This measure captured the father/male guardian’s education and occupation, the mother/female guardian’s education and occupation, and the household income. Higher scores reflect higher levels of educational attainment, occupational prestige, and income. In the present analyses, the socioeconomic composite index was categorized into quintiles and further divided into the lowest first and second quintiles, versus the third, fourth and fifth quintiles.

Two variables measured the school-level environment: percentage of students eligible for free school meals, and percentage of students from a racial/ethnic background other than White non-Hispanic. These two variables were dichotomized as more than or equal to 50% of students belonging to each category. Both variables were measured in the kindergarten, first, third, fifth and eighth grade data collections.

To capture the neighborhood environment, a variable was included which measured the level of safety of the neighborhood in kindergarten, first, third, fifth and eighth grades. Parents were asked “How safe is it for children to play outside during the day in your neighborhood?” with responses ranging from 1, not at all safe, to 3, very safe. For the present analyses, response categories were recoded into 1 “not at all and somewhat safe,” and 0 “very safe.”

#### Predictor Variables

The race/ethnicity and gender of the children were assessed during the parent interview. In order to empirically measure the intersection between race/ethnicity and gender in the classes of disadvantage, a set of six dummy variables were created that combined racial/ethnic and gender categories into White boys, White girls, Black boys, Black girls, Latino boys, and Latina girls.

### Statistical Analyses

This study used the manual 3-step approach in mixture modeling with auxiliary variables [64, 65] to independently evaluate the relationship between the predictor auxiliary variables (the combined race/ethnicity and gender groups), the latent class variable of advantage/disadvantage, and the outcome (non-cognitive skills, socioemotional development, cognitive assessments). This is a data-driven, mixture modelling technique which uses indicator variables (in this case the variables described under Individual and Contextual Disadvantage Variables section) to identify a number of latent classes. It also includes auxiliary information in the form of covariates (the race/ethnicity and gender combinations described under Predictor Variables) and distal outcomes (the eight outcome variables), to better explore the relationships between the characteristics that make up the latent classes, the predictors of class membership, and the associated consequences of membership into each class.

The first step in the 3-step procedure is to estimate the measurement part of the joint model (i.e., the latent class model) by creating the latent classes without adding covariates. Latent class analyses first evaluated the fit of a 2-class model, and systematically increased the number of classes in subsequent models until the addition of latent classes did not further improve model fit. For each model, replication of the best log-likelihood was verified to avoid local maxima. To determine the optimal number of classes, models were compared across several model fit criteria. First, the sample-size adjusted Bayesian Information Criterion (BIC) [66] was evaluated; lower relative BIC values indicate improved model fit. Given that the BIC criterion tends to favor models with fewer latent classes [67], the Lo, Mendell, and Rubin likelihood ratio test (LMR-LRT) statistic [68] was also considered. The LMR-LRT can be used in mixture modeling to compare the fit of the specified class solution (*k*-class model) to a model with fewer classes (*k*-1 class model). A non-significant chi-square value suggests that a model with one fewer class is preferred. Entropy statistics, which measure the separation of the classes based on the posterior class membership probabilities, were also examined; entropy values approaching 1 indicate clear separation between classes [69].

After determining the latent class model in step 1, the second step of the analyses used the latent class posterior distribution to generate a nominal variable *N*, which represented the most likely class [64]. During the third step, the measurement error for *N* was accounted for while the model was estimated with the outcomes and predictor auxiliary variables [64]. The last step of the analysis examined whether race/ethnic and gender categories predict class membership, and whether class membership predicts the outcomes of interest.

All analyses were conducted using MPlus v. 7.11 [70], and used longitudinal weights to account for differential probabilities of selection at each sampling stage and to adjust for the effects of non-response. A robust standard error estimator was used in MPlus to account for the clustering of observations in the ECLS-K.

## Results

Four distinct classes of advantage/disadvantage were identified in the latent class analysis (see Table 1).

**Table 1:** Fit indices of Latent Class Analysis.

Number of classes	Sample Size Adjusted BIC	Entropy	Loglikelihood	Vuong-Lo-Mendell-Rubin Likelihood Ratio Test (LMR-LRT) Δ	*p* Δ
2	321340.907	0.948	-160413.583		
3	302819.304	0.962	-151022.835	18781.496	0.0000
4	291380.091	0.957	-145173.282	11699.106	0.0054
5	285648.669	0.956	-142177.625	5991.314	0.5906

Class characteristics are shown in Table A in S1 File. Trajectories of advantage and disadvantage were stable across ECLS-K waves, so that none of the classes identified changed in individual and contextual characteristics across time. The largest proportion of the sample (47%; Class 3: Individually and Contextually Wealthy) lived in individual and contextual privilege, with very low proportions of children in socioeconomic deprived contexts. A class representing the opposite characteristics (children living in individually- and contextually-deprived circumstances) was also identified in the analyses (19%; Class 1: Individually and Contextually Disadvantaged). Class 1 had the highest proportion of children living in socioeconomic deprivation, attending schools with more than 50% racial/ethnic minority students, and living in unsafe neighborhoods, but did not have a high proportion of children with the lowest parental expectations. Class 4 (19%; Individually Disadvantaged, Contextually Wealthy) had the highest proportion of children with the lowest parental expectations (parents reporting across waves that they expected children to achieve up to a high school education). Class 4 (Individually Disadvantaged, Contextually Wealthy) also had high proportions of children living in individual-level socioeconomic deprivation, but had low proportions of children attending a school with over 50% of children eligible for free school meals. It also had relatively low proportions of children living in unsafe neighborhoods and low proportions of children attending diverse schools, forming a class with a mixture of individual-level deprivation, and contextual-level advantage. The last class was composed of children who lived in individually-wealthy environments, but who also lived in unsafe neighborhoods and attended diverse schools where more than 50% of pupils were eligible for free school meals (13%; Class 2: Individually Wealthy, Contextually Disadvantaged; see Table A in S1 File).

The combined intersecting racial/ethnic and gender characteristics yielded six groups consisting of White boys (n = 2998), White girls (n = 2899), Black boys (n = 553), Black girls (n = 560), Latino boys (n = 961), and Latina girls (n = 949). All pairs containing at least one minority status of either race/ethnicity or gender (e.g., Black boys, Black girls, Latino boys, Latina girls) were more likely than White boys to be assigned to the more disadvantaged classes, as compared to being assigned to Class 3, the least disadvantaged (see Table B in S1 File).

### Racial/Ethnic and Gender Differences in Eighth-Grade Academic Outcomes

Table 2 shows broad patterns of intersecting racial/ethnic and gender inequalities in academic outcomes, although interesting differences emerge across racial/ethnic and gender groups. Whereas Black boys achieved lower scores than White boys across all classes on the math, reading and science assessments, this was not the case for Latino boys, who only underperformed White boys on the science assessment within the most privileged class (Class 3: Individually and Contextually Wealthy). Latina girls, in contrast, outperformed White boys on reading scores within Class 4 (Individually Disadvantaged, Contextually Wealthy), but scored lower than White boys on science and math assessments, although only when in the two most privileged classes (Class 3 and 4). For Black girls the effect of class membership was not as pronounced, and they had lower science and math scores than White boys across all but one instance.

**Table 2:** Within-class differences in academic outcomes across racial/ethnic and gender groups.

	Class 1: Individually and Contextually Disadvantaged	Class 2: Individually Wealthy, Contextually Disadvantaged	Class 3: Individually and Contextually Wealthy	Class 4: Individually Disadvantaged, Contextually Wealthy
	Coeff (S.E.)	Coeff (S.E.)	Coeff (S.E.)	Coeff (S.E.)
**Math Scores**				
White boys	Reference	Reference	Reference	Reference
White girls	2.46 (2.59)	0.08 (2.16)	-1.05 (0.52)*	0.87 (1.08)
Black boys	-4.74 (1.62)***	-4.58 (2.08)*	-4.76 (2.34)*	-2.95 (1.66)
Black girls	-4.04 (1.51)**	-6.09 (1.69)***	-6.51 (1.64)***	-4.75 (2.61)
Hispanic boys	0.54 (1.42)	-2.84 (1.61)	-1.98 (1.44)	-0.84 (1.46)
Hispanic girls	-0.13 (1.41)	-3.55 (1.30)**	-2.06 (1.37)	1.69 (1.52)
**Reading Scores**				
White boys	Reference	Reference	Reference	Reference
White girls	4.80 (3.57)	0.83 (2.54)	1.91 (0.50)***	2.90 (1.07)**
Black boys	-4.57 (1.17)***	-3.50 (1.65)*	-5.73 (2.18)**	-4.42 (1.64)**
Black girls	-1.41 (1.38)	-3.57 (1.45)**	-3.01 (1.93)	-0.40 (1.19)
Hispanic boys	-0.13 (1.07)	-2.90 (1.63)	-2.19 (1.61)	-2.25 (1.58)
Hispanic girls	1.79 (1.14)	-0.79 (1.25)	-0.14 (1.36)	2.32 (1.20)*
**Science Scores**				
White boys	Reference	Reference	Reference	Reference
White girls	2.49 (2.26)	-2.19 (2.22)	-2.03 (0.46)***	-0.33 (1.03)
Black boys	-3.93 (1.34)***	-4.58 (1.70)**	-6.27 (2.10)***	-5.41 (1.65)***
Black girls	-5.26 (1.33)***	-6.52 (1.70)***	-11.24 (1.50)***	-7.52 (2.18)***
Hispanic boys	0.69 (1.16)	-2.80 (1.92)	-2.39 (1.17)*	-2.17 (1.82)
Hispanic girls	0.62 (1.17)	-4.29 (1.30)***	-4.73 (1.24)***	-1.85 (1.41)

*p<0.05,

**p<0.01,

***p<0.001; statistical significance at α = 0.05.

In general, the largest inequalities in academic outcomes across racial/ethnic and gender groups appeared in the most privileged classes. For example, results show no differences in math scores across racial/ethnic and gender categories within Class 4, the most disadvantaged class, but in all other classes that contain an element of advantage, and particularly in Class 3 (Individually and Contextually Wealthy), there are large gaps in math scores across racial/ethnic and gender groups, when compared to White boys. These patterns of heightened inequality in the most advantaged classes are similar for reading and science scores (see Table 2).

### Racial/Ethnic and Gender Differences in Eighth-Grade Non-Academic Outcomes

Interestingly, racialized and gendered patterns of inequality observed in academic outcomes were not as stark in non-cognitive academic outcomes (see Table 3).

**Table 3:** Within-class differences in non-academic outcomes across racial/ethnic and gender groups.

	Class 1: Individually and Contextually Disadvantaged	Class 2: Individually Wealthy, Contextually Disadvantaged	Class 3: Individually and Contextually Wealthy	Class 4: Individually Disadvantaged, Contextually Wealthy
	Coeff (S.E.)	Coeff (S.E.)	Coeff (S.E.)	Coeff (S.E.)
**Perceived Interest/Competence, Reading**				
White boys	Reference	Reference	Reference	Reference
White girls	0.16 (0.39)	0.17 (0.17)	0.37 (0.04)***	0.42 (0.08)***
Black boys	0.16 (0.15)	-0.08 (0.16)	-0.50 (0.15)***	-0.13 (0.17)
Black girls	0.29 (0.15)	0.28 (0.15)	0.22 (0.22)	0.31 (0.24)
Hispanic boys	0.01 (0.13)	-0.24 (0.12)*	-0.17 (0.09)	-0.14 (0.09)
Hispanic girls	0.13 (0.14)	0.18 (0.12)	0.26 (0.08)***	0.17 (0.17)
**Perceived Interest/Competence, Math**				
White boys	Reference	Reference	Reference	Reference
White girls	-0.05 (0.54)	-0.17 (0.17)	-0.01 (0.05)	0.10 (0.11)
Black boys	-0.19 (0.18)	0.03 (0.16)	0.19 (0.22)	0.07 (0.21)
Black girls	-0.43 (0.19)*	-0.26 (0.19)	0.13 (0.26)	-0.33 (0.19)
Hispanic boys	-0.20 (0.15)	0.01 (0.14)	-0.07 (0.09)	0.01 (0.13)
Hispanic girls	-0.51 (0.16)***	-0.16 (0.15)	-0.39 (0.13)***	0.03 (0.17)
**Internalizing Behavior**				
White boys	Reference	Reference	Reference	Reference
White girls	-0.08 (0.26)	0.14 (0.10)	0.08 (0.03)***	0.16 (0.06)*
Black boys	0.06 (0.12)	-0.23 (0.09)**	0.08 (0.20)	-0.21 (0.10)*
Black girls	0.14 (0.10)	-0.09 (0.10)	-0.24 (0.14)	0.12 (0.15)
Hispanic boys	0.04 (0.09)	0.10 (0.10)	-0.06 (0.06)	-0.02 (0.14)
Hispanic girls	0.22 (0.09)**	0.09 (0.08)	0.18 (0.08)*	-0.10 (0.09)
**Locus of Control**				
White boys	Reference	Reference	Reference	Reference
White girls	0.37 (0.21)	0.08 (0.13)	0.13 (0.03)***	0.06 (0.07)
Black boys	0.09 (0.15)	0.17 (0.13)	-0.05 (0.20)	0.13 (0.13)
Black girls	-0.15 (0.13)	0.14 (0.09)	0.25 (0.13)	-0.21 (0.17)
Hispanic boys	0.01 (0.12)	0.08 (0.09)	-0.02 (0.08)	-0.07 (0.12)
Hispanic girls	0.10 (0.12)	0.13 (0.09)	-0.03 (0.10)	0.06 (0.10)
**Self-Concept**				
White boys	Reference	Reference	Reference	Reference
White girls	0.24 (0.31)	-0.11 (0.18)	-0.04 (0.04)	0.11 (0.08)
Black boys	0.25 (0.13)*	0.25 (0.12)*	0.08 (0.22)	0.34 (0.15)*
Black girls	0.10 (0.13)	0.30 (0.11)**	0.37 (0.13)***	-0.24 (0.26)
Hispanic boys	0.08 (0.11)	-0.02 (0.10)	-0.04 (0.08)	-0.03 (0.11)
Hispanic girls	-0.03 (0.11)	-0.04 (0.09)	-0.24 (0.12)	0.08 (0.13)

*p<0.05,

**p<0.01,

***p<0.001; statistical significance at α = 0.05.

Racial/ethnic and gender differences were small across socioemotional outcomes, and in fact, White boys were outperformed on several outcomes. Black boys scored lower than White boys on internalizing behavior and higher on self-concept within Classes 2 (Individually Wealthy, Contextually Disadvantaged) and 4 (Individually Disadvantaged, Contextually Wealthy), and Black girls scored higher than White boys on self-concept within Classes 2 and 3 (Individually Wealthy, Contextually Disadvantaged, and Individually and Contextually Wealthy, respectively). White and Latina girls, but not Black girls, scored higher than White boys on internalizing behavior (within Classes 3 and 4 for White girls, and within Classes 1 and 3 for Latina girls; see Table 3).

As with academic outcomes, most racial/ethnic and gender differences also emerged within the most privileged classes, and particularly in Class 3 (Individually and Contextually Wealthy), although in the case of perceived interest/competence in reading, White and Latina girls performed better than White boys. White girls also reported higher perceived interest/competence in reading than White boys in Class 4: Individually Disadvantaged, Contextually Wealthy.

## Discussion

This study set out to examine inequalities across several eighth grade academic and non-academic outcomes at the intersection of race/ethnicity, gender, and socioeconomic status. It first identified four classes of longstanding individual- and contextual-level disadvantage; then determined membership to these classes depending on racial/ethnic and gender groups; and finally compared non-cognitive skills, academic assessment scores, and socioemotional outcomes across intersecting gender, racial/ethnic and socioeconomic social positions.

Results show the clear influence of race/ethnicity in determining membership to the most disadvantaged classes. Across gender dichotomies, Black students were more likely than White boys to be assigned to all classes of disadvantage as compared to the most advantaged class, and this was particularly strong for the most disadvantaged class, which included elements of both individual- and contextual-level disadvantage. Latino boys and girls were also more likely than White boys to be assigned to all the disadvantaged classes, but the strength of the association was much smaller than for Black students. Whereas membership into classes of disadvantage appears to be more a result of structural inequalities strongly driven by race/ethnicity, the salience of gender is apparent in the distribution of academic assessment outcomes within classes of disadvantage. Results show a gendered pattern of math, reading and science assessments, particularly in the most privileged class, where girls from all ethnic/racial groups (although mostly from Black and Latino racial/ethnic groups) underperform White boys in math and science, and where Black boys score lower, and White girls higher, than White boys in reading.

With the exception of educational assessments, gender and racial/ethnic inequalities *within* classes are either not very pronounced or in the opposite direction (e.g. racial/ethnic and gender minorities outperform White males), but differences in outcomes *across* classes are stark. The strength of the association between race/ethnicity and class membership, and the reduced racial/ethnic and gender inequalities within classes of advantage and disadvantage, attest to the importance of socioeconomic status and wealth in explaining racial/ethnic inequalities; should individual and contextual disadvantage be comparable across racial/ethnic groups, racial/ethnic inequalities would be substantially reduced. This being said, most within-class differences were observed in the most privileged classes, showing that benefits brought about by affluence and advantage are not equal across racial/ethnic and gender groups. The measures of advantage and disadvantage captured in this study relate to characteristics afforded by parental resources, implying an intergenerational transmission of disadvantage, regardless of the presence of absolute adversity in childhood. This pattern of differential returns of affluence has been shown in other studies, which report that White teenagers benefit more from the presence of affluent neighbors than do Black teenagers [71]. Among adult populations, studies show that across several health outcomes, highly educated Black adults fare worse than White adults with the lowest education [72]. Intersectional approaches such as the one applied in this study reveal how power within gendered and racialized institutional settings operates to undermine access to and use of resources that would otherwise be available to individuals of advantaged classes [72]. The present study further contributes to this literature by documenting how, in a key stage of the life course, similar levels of advantage, but not disadvantage, lead to different academic outcomes across racial/ethnic and gender groups. These findings suggest that, should socioeconomic inequalities be addressed, and levels of advantage were similar across racial/ethnic and gender groups, systems of oppression that pattern the racialization and socialization of children into racial/ethnic and gender roles in society would still ensure that inequalities in academic outcomes existed across racial/ethnic and gender categories. In other words, racism and sexism have a direct effect on academic and non-academic outcomes among 8^th^ graders, independent of the effect of socioeconomic disadvantage on these outcomes. An important limitation of the current study is that although it uses a comprehensive measure of advantage/disadvantage, including elements of deprivation and affluence at the family, school and neighborhood levels through time, it failed to capture these two key causal determinants of racial/ethnic and gender inequality: experiences of racial and gender discrimination.

Despite this limitation, it is important to note that socioeconomic inequalities in the US are driven by racial and gender bias and discrimination at structural and individual levels, with race and gender discrimination exerting a strong influence on academic and non-academic inequalities. Racial discrimination, prevalent in the US and in other industrialized nations [38, 73] determines differential life opportunities and resources across racial/ethnic groups, and is a crucial determinant of racial/ethnic inequalities in health and development throughout life and across generations [37, 38]. In the context of this study’s primary outcomes within school settings, racism and racial discrimination experienced by both the parents and the children are likely to contribute towards explaining observed racial/ethnic inequalities in outcomes within classes of disadvantage. Gender discrimination—another system of oppression—is apparent in this study in relation to academic subjects socially considered as typically male or female orientated. For example, results show no difference between Black girls and White boys from the most advantaged class in terms of perceived interest and competence in math but, in this same class, Black girls score much lower than White boys in the math assessment. This difference, not explained by intrinsic or socioeconomic differences, can be contextualized as a consequence of experienced intersecting racial and gender discrimination. The consequences of the intersection between two marginalized identities are found throughout the results of this study when comparing across broad categorizations of race/ethnicity and gender, and in more detailed conceptualizations of minority status. Growing up Black, Latino or White in the US is not the same for boys and girls, and growing up as a boy or a girl in America does not lead to the same outcomes and opportunities for Black, Latino and White children as they become adults. With this study’s approach of intersectionality one can observe the complexity of how gender and race/ethnicity intersect to create unique academic and non-academic outcomes. This includes the contrasting results found for Black and Latino boys, when compared to White boys, which show very few examples of poorer outcomes among Latino boys, but several instances among Black boys. Results also show different racialization for Black and Latina girls. Latina girls, but not Black girls, report higher internalizing behavior than White boys, whereas Black girls, but not Latina girls, report higher self-concept than White boys. Black boys also report higher self-concept and lower internalizing behavior than White boys, findings that mirror research on self-esteem among Black adolescents [74, 75]. In cognitive assessments, intersecting racial/ethnic and gender differences emerge across classes of disadvantage. For example, Black girls in all four classes score lower on science scores than White boys, but only Latina girls in the most advantaged class score lower than White boys. Although one can observe differences in the racialization of Black and Latino boys and girls across classes of disadvantage, findings about broad differences across Latino children compared to Black and White children should be interpreted with caution. The Latino ethnic group is a large, heterogeneous group, representing 16.7% of the total US population [76]. The Latino population is composed of a variety of different sub-groups with diverse national origins and migration histories [77], which has led to differences in sociodemographic characteristics and lived experiences of ethnicity and minority status among the various groups. Differences across Latino sub-groups are widely documented, and pooled analyses such as those reported here are masking differences across Latino sub-groups, and providing biased comparisons between Latino children, and Black and White children.

Poorer performance of girls and racial/ethnic minority students in science and math assessments (but not in self-perceived competence and interest) might result from stereotype threat, whereby negative stereotypes of a group influence their member’s performance [78]. Stereotype threat posits that awareness of a social stereotype that reflects negatively on one's social group can negatively affect the performance of group members [35]. Reduced performance only occurs in a threatening situation (e.g., a test) where individuals are aware of the stereotype. Studies show that early adolescence is a time when youth become aware of and begin to endorse traditional gender and racial/ethnic stereotypes [79]. Findings among youth parallel findings among adult populations, which show that adult men are generally perceived to be more competent than women, but that these perceptions do not necessarily hold for Black men [80]. These stereotypes have strong implications for interpersonal interactions and for the wider structuring of systemic racial/ethnic and gender inequalities. An example of the consequences of negative racial/ethnic and gender stereotypes as children grow up is the well-documented racial/ethnic and gender pay gap: women earn less than men [81], and racial/ethnic minority women and men earn less than White men [82].

In addition to the focus on intersectionality, a strength of this study is its person-centered methodological approach, which incorporates measures of advantage and disadvantage across individual and contextual levels through nine years of children’s socialization. Children live within multiple contexts, with risk factors at the family, school, and neighborhood level contributing to their development and wellbeing. Individual risk factors seldom operate in isolation [83], and they are often strongly associated both within and across levels [84]. All risk factors captured in the latent class analyses have been independently associated with increased risk for academic problems [10, 71, 85, 86], and given that combinations of risk factors that cut across multiple domains explain the association between early risk and later outcomes better than any isolated risk factor [83, 84], the incorporation of person-centered and intersectionality approaches to the study of racial/ethnic, gender, and socioeconomic inequalities across school outcomes provides new insight into how children in marginalized social groups are socialized in the early life course.

## Conclusions

The contrasting outcomes between racial/ethnic and gender minorities in self-assessment and socioemotional outcomes, as compared to standardized assessments, provide support for the detrimental effect that intersecting racial/ethnic and gender discrimination have in patterning academic outcomes that predict success in adult life. Interventions to eliminate achievement gaps cannot fully succeed as long as social stratification caused by gender and racial discrimination is not addressed [87, 88].

## Supporting Information

S1 File Supporting Tables. **Table A:** Class characteristics. **Table B:** Associations between race/ethnicity and gender groups and assigned class membership (membership to Classes 1, 2 or 4 as compared to Class 3: Individually and Contextually Wealthy).
(DOCX)
