# Supplementary material for: Understanding the Influence of Race/Ethnicity, Gender, and Class on Inequalities in Academic and Non-Academic Outcomes among Eighth-Grade Students: Findings from an Intersectionality Approach
Source: PLoS One. 2015 Oct 27;10(10):e0141363. doi: 10.1371/journal.pone.0141363 (PMC4624767; doi:10.1371/journal.pone.0141363)
Supplement: S1 File — Table A: Class characteristics. Table B: Associations between race/ethnicity and gender groups and assigned class membership (membership to Classes 1, 2 or 4 as compared to Class 3: Individually and Contextually Wealthy). (DOCX) [file pone.0141363.s001.docx]

**S1 Table A. Class characteristics**

| **Observed Variables** | **Overall proportion** | **CLASS 1** | **CLASS 2** | **CLASS 3** | **CLASS 4** |
| --- | --- | --- | --- | --- | --- |
|  |  | **Individually and Contextually Disadvantaged** | **Individually Wealthy, Contextually Disadvantaged** | **Individually and Contextually Wealthy** | **Individually Disadvantaged, Contextually Wealthy** |
|  | N=10115 | N=1956 | N=1443 | N=4798 | N=1918 |
| Lowest SES Kind. | 0.362 | 0.890 | 0.186 | 0.040 | 0.760 |
| Lowest SES 1^st^ G. | 0.346 | 0.880 | 0.132 | 0.022 | 0.774 |
| Lowest SES 3^rd^ G. | 0.356 | 0.919 | 0.102 | 0.015 | 0.826 |
| Lowest SES 5^th^ G. | 0.345 | 0.931 | 0.079 | 0.008 | 0.787 |
| Lowest SES 8^th^ G. | 0.372 | 0.914 | 0.122 | 0.050 | 0.811 |
| Single mom Kind. | 0.195 | 0.425 | 0.204 | 0.055 | 0.304 |
| Single mom 1^st^ G. | 0.205 | 0.424 | 0.192 | 0.073 | 0.324 |
| Single mom 3^rd^ G. | 0.211 | 0.406 | 0.185 | 0.087 | 0.347 |
| Single mom 5^th^ G. | 0.219 | 0.416 | 0.177 | 0.098 | 0.354 |
| Single mom 8^th^ G. | 0.222 | 0.399 | 0.178 | 0.114 | 0.347 |
| Food insecurity K. | 0.086 | 0.199 | 0.035 | 0.021 | 0.174 |
| Food insecurity 1^st^ | 0.073 | 0.193 | 0.039 | 0.012 | 0.132 |
| Food insecurity 2^nd^ | 0.099 | 0.250 | 0.073 | 0.015 | 0.175 |
| Food insecurity 3^rd^ | 0.097 | 0.194 | 0.059 | 0.027 | 0.206 |
| Below poverty threshold Kinderg. | 0.195 | 0.624 | 0.072 | 0.016 | 0.298 |
| Below poverty threshold 5^th^ grade | 0.194 | 0.611 | 0.047 | 0.006 | 0.352 |
| Below poverty threshold 8^th^ grade | 0.177 | 0.540 | 0.046 | 0.009 | 0.326 |
| Parental expectation up to high school Kind. | 0.092 | 0.164 | 0.036 | 0.029 | 0.219 |
| Parental expectation up to high school 1^st^ G | 0.102 | 0.189 | 0.043 | 0.027 | 0.249 |
| Parental expectation up to high school 3^rd^ G | 0.095 | 0.156 | 0.038 | 0.029 | 0.242 |
| Parental expectation up to high school 5^th^ G | 0.097 | 0.154 | 0.029 | 0.026 | 0.269 |
| Parental expectation up to high school 8^th^ G. | 0.086 | 0.145 | 0.025 | 0.021 | 0.236 |
| Moved since birth Kindergarten | 0.276 | 0.333 | 0.273 | 0.223 | 0.353 |
| Moved since last interview 1^st^ grade | 0.027 | 0.048 | 0.021 | 0.015 | 0.040 |
| Moved since last interview 3^rd^ grade | 0.021 | 0.032 | 0.011 | 0.018 | 0.024 |
| Moved since last interview 5^th^ grade | 0.015 | 0.008 | 0.011 | 0.011 | 0.037 |
| Moved since last interview 8^th^ grade | 0.029 | 0.021 | 0.020 | 0.025 | 0.053 |
| 50%+ pupils eligible for Free School Meal Kindergarten | 0.301 | 0.757 | 0.461 | 0.049 | 0.212 |
| 50%+ pupils eligible for Free School Meal 1^st^ grade | 0.281 | 0.741 | 0.398 | 0.041 | 0.201 |
| 50%+ pupils eligible for Free School Meal 3^rd^ grade | 0.303 | 0.790 | 0.468 | 0.044 | 0.215 |
| 50%+ pupils eligible for Free School Meal 5^th^ grade | 0.320 | 0.783 | 0.522 | 0.067 | 0.213 |
| 50%+ pupils eligible for Free School Meal 8^th^ grade | 0.249 | 0.640 | 0.395 | 0.045 | 0.195 |
| 50%+ racial/ethnic minorities in school Kindergarten | 0.338 | 0.971 | 0.817 | 0.049 | 0.040 |
| 50%+ racial/ethnic minorities in school 1^st^ grade | 0.342 | 0.973 | 0.854 | 0.037 | 0.050 |
| 50%+ racial/ethnic minorities in school 3^rd^ grade | 0.351 | 0.948 | 0.906 | 0.038 | 0.073 |
| 50%+ racial/ethnic minorities in school 5^th^ grade | 0.358 | 0.951 | 0.892 | 0.046 | 0.111 |
| 50%+ racial/ethnic minorities in school 8^th^ grade | 0.358 | 0.911 | 0.800 | 0.096 | 0.133 |
| Neighborhood unsafe Kinder. | 0.304 | 0.587 | 0.404 | 0.152 | 0.319 |
| Neighborhood unsafe 1^st^ grade | 0.277 | 0.549 | 0.415 | 0.127 | 0.274 |
| Neighborhood unsafe 3^rd^ grade | 0.234 | 0.502 | 0.317 | 0.091 | 0.254 |
| Neighborhood unsafe 5^th^ grade | 0.236 | 0.540 | 0.308 | 0.079 | 0.270 |
| Neighborhood unsafe 8^th^ grade | 0.221 | 0.517 | 0.287 | 0.072 | 0.245 |

**S1 Table B. Associations between race/ethnicity and gender groups and assigned class membership (membership to Classes 1, 2 or 4 as compared to Class 3: Individually and Contextually Wealthy)**

|  | Coeff. (S.E.) |
| --- | --- |
| Class 1: **Individually and Contextually Disadvantaged** |  |
| White boys | Reference |
| White girls | -1.27 (0.37)*** |
| Black boys | 4.34 (0.37)*** |
| Black girls | 4.05 (0.33)*** |
| Hispanic boys | 3.09 (0.20)*** |
| Hispanic girls | 3.17 (0.21)*** |
| Class 2: **Individually Wealthy, Contextually Disadvantaged** |  |
| White boys | Reference |
| White girls | -0.65 (0.20)*** |
| Black boys | 3.54 (0.38)*** |
| Black girls | 3.12 (0.34)*** |
| Hispanic boys | 1.59 (0.22)*** |
| Hispanic girls | 1.87 (0.21)*** |
| Class 4: **Individually Disadvantaged, Contextually Wealthy** |  |
| White boys | Reference |
| White girls | 0.02 (0.13) |
| Black boys | 2.26 (0.40)*** |
| Black girls | 1.52 (0.39)*** |
| Hispanic boys | 0.50 (0.23)* |
| Hispanic girls | 0.58 (0.25)* |

*p<0.05, **p<0.01, ***p<0.001; statistical significance at α=0.05
